# Failure to awaken from general anesthesia due to infratentorial hemorrhage after cervical spine surgery: A case report

**DOI:** 10.1097/MD.0000000000017678

**Published:** 2019-11-01

**Authors:** Ji Hyun Kim, Yehun Jin, Seong Wook Hong

**Affiliations:** Department of Anesthesiology and Pain Medicine, Kyungpook National University Hospital, Daegu, Republic of Korea.

**Keywords:** emergence, general anesthesia, infratentorial hemorrhage, prolonged coma

## Abstract

**Rationale:**

Emergence is not simply the reverse process of induction. Many dynamic situations could occur in this period by distinct neurobiology as recent studies indicated. Herein we report a rare case of failure of emergence from general anesthesia after cervical spine surgery.

**Patient concerns:**

Despite the perioperative vital signs and laboratory results were unremarkable, the patient could not recover his mental status and spontaneous breathing during emergence. 20 minutes after cessation of anesthetic drug administration, his blood pressure suddenly began to decrease requiring transfusion and vasopressor.

**Diagnosis:**

After thorough inspection of intraoperative alterations of hemodynamic and metabolic values, which showed no significant changes except possible signs of delayed volume loss, cerebrovascular bleeding was most suspected as the cause of the event. Computed tomography was performed and infratentorial hemorrhage after cervical spine surgery was checked.

**Interventions:**

Decompression operation was required for removing the hemorrhage. However, the patient's family refused further management considering his limited life expectancy.

**Outcomes:**

The patient expired on postoperative day 5.

**lessons:**

Failure to awaken is a relatively rare event. It could be confused with simple delayed emergence, which is often caused by residual drug effect. However, when it occurs, the result could be devastating. Therefore, appropriate recognition and prompt response are required to decrease the mortality and morbidity of the patient.

## 1 Introduction

Emergence from general anesthesia refers to the wake-up period after withdrawal of general anesthetic drug administration. Over the past decade, there has been growing interest in understanding this process.^[1–3]^ As Hight et al^[1]^ reported, emergence is not simply an opposite mirror image of the induction period. It is very much a critical and active process based on distinct neurobiology, and various undesirable complications, including hypertension, tachycardia, coughing, laryngospasm, and delirium, may occur. Prolonged recovery of consciousness is also relatively common, and is often caused by residual anesthetic drug effects. Compared with delayed emergence, failure to awaken is rarely reported. However, aside from drug effects, physicians should be aware of other metabolic and surgical events that could affect the emergence process. Otherwise, possible causes of failure to awaken could be overlooked. Therefore, to minimize patient distress, it is important for anesthesiologists to identify and respond appropriately when recovery is prolonged. We report a rare case of failure to awaken, which was revealed to be caused by remote infratentorial hemorrhage after spinal surgery.

## 2 Case report

A 167 cm, 53 kg, 63-year-old man was scheduled for occipitocervical fusion with screw fixation. He underwent a left upper lobectomy 1 year ago due to lung cancer, and underwent 4 cycles of postoperative radiotherapy and chemotherapy. However, he recently exhibited metastasis and pathological fractures on C1 and C2. He has been undergoing anticoagulation therapy until 10 days before the operation because he suffered right renal infarction related to cancer emboli 1 year ago. Follow-up renal function tests and computed tomography (CT) before the operation revealed recovered perfusion, and urine output of ≥3 L/d was observed. His routine laboratory investigations revealed hyponatremia, with ionized sodium concentration of 125 mM, which was suspected to be related to paraneoplastic syndrome of inappropriate secretion of antidiuretic hormone. The patient had mild fatigue but, showed alert mental status with normal motor function. Other complication related with chronic hyponatremia was not checked. The patient also had a history of pneumonia, which was developed 1 month previously. Consolidation in both lower lung fields and multifocal areas of reticular opacity were checked on preoperative chest radiograph and CT. Arterial blood gas analysis (ABGA) performed on room air showed partial pressure of carbon dioxide, partial pressure of oxygen, and oxygen saturation (SpO_2_) of 31.3 mm Hg, 75.6 mm Hg and 96.5%, respectively. Although pulmonary function test was not available due to his severe neck pain, the patient was considered to be much improved from pneumonia, reflected by the lack of significant respiratory distress. Other than this, his routine perioperative results were unremarkable.

Induction and maintenance of general anesthesia were achieved by total intravenous anesthesia using an infusion device (Orchestra Base Primea, Fresenius Kabi, Brezins, France) for effect-site target-controlled infusion (TCI) of propofol and remifentanil. Rocuronium was administered as a neuromuscular blocking agent. Considering the instability of the patient's cervical spine, a flexible fiberoptic bronchoscope with an external diameter of 4.1 mm (PortaView, LF-GP; Olympus, Tokyo, Japan) was used for intubation. Standard monitoring, which includes noninvasive blood pressure (BP), electrocardiography (ECG), oximetry, and bispectral index (BIS; Model A-3000 vista with Sensor Bis quatro, Aspect Medical Systems, Norwood, MA), was applied. For further monitoring of hemodynamic alterations, a Vigileo monitor with FloTrac sensor and PreSep catheter (Edwards Lifesciences LLC; One Edwards Way, Irvine, CA) were cannulated via both right radial artery and internal jugular vein.

**Figure 1:**
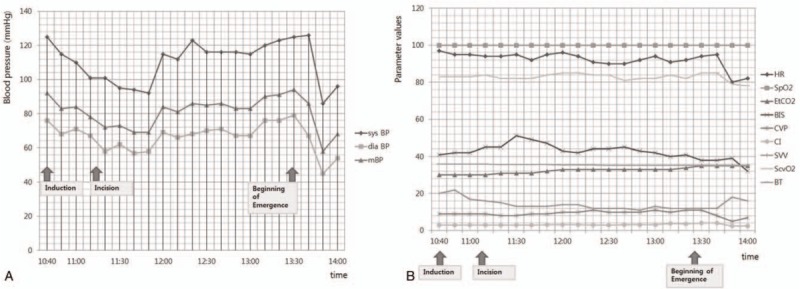
Intraoperative changes in hemodynamic values of the patient. ^∗^Parameter values in (B) are measured by bmp for HR, % for SpO2, mm Hg for EtCO2 and CVP, L/min/m^2^ for CI, % for SVV and ScvO_2_, °C for BT. BIS = bispectral index, BT = body temperature CI = cardiac index, CVP = central venous pressure, dia BP = diastolic blood pressure, EtCO_2_ = end-tidal carbon dioxide concentration, HR = heart rate, mBP = mean blood pressure, ScvO_2_ = central venous oxygen saturation, SpO_2_ = oxygen saturation, SVV = stroke volume variation, sys BP = systolic blood pressure.

The patient's initial ECG demonstrated sinus rhythm with a slightly fast heart rate (HR) showing 97 bpm, which the authors suspected was caused by pain or dehydration related to his chronic illness. Other vital signs were unremarkable, with BP, SpO_2,_ and body temperature (BT) of 125/76 mm Hg, 100%, and 36.0°C, respectively. Baseline values measured from both arterial and central catheterization revealed central venous pressure (CVP), cardiac index (CI), stroke volume variation (SVV), and central venous oxygen saturation (ScvO_2_) of 9 mm Hg, 2.8 L/min/m^2^, 20%, and 83%, respectively. For laboratory checkup, ABGA was performed in the operating room. Initial result showed hematocrit (Hct), ionized sodium concentration of 26%, 123 mM and otherwise unremarkable. During the operation, 2.3 L of crystalloid fluid (Plasma solution A, CJ Pharma, Seoul, Korea) and 0.4 L of hydroxyethyl starch (Volulyte 6%, Fresenius Kabi Deutschland GmbH, Germany) were administered for volume replacement. Five hundred milliliters of packed red blood cells (PRBC) were transfused based on initial Hct, the general condition of the patient, and intraoperative blood loss. Urine output was checked, and was, on average, approximately 200 mL/h. During the operation, his vital signs and laboratory results showed no significant changes compared with baseline values, and remained stable when the emergence period started (Fig. 1). Although Hct decreased to 22% in the middle of the operation, ABGA performed immediately before the emergence period revealed 25%, and was expected to further increase. Because the blood sample was checked during transfusion of the second 250 mL of PRBC and the operation approached its conclusion without any additional bleeding.

Ten minutes after cessation of anesthetic drug administration, the patient demonstrated no signs of recovery. Vital signs at this time showed BP, HR, SpO_2_, end-tidal carbon dioxide concentration, and BT of 126/67 mm Hg, 95 bpm, 100%, 35 mm Hg, and 35.2°C, respectively. Vigileo monitor with FloTrac sensor and PreSep catheter showed CI, SVV, and ScvO_2_ values of 4.1 L/min/m^2^, 12%, and 85% respectively. Given that the perioperative hemodynamic values were stable, a residual anesthetic drug effect appeared to be the most likely the cause of delayed emergence at this point. Fifteen minutes later, despite plasma and effect side concentration (plasma concentration [Cp] and effect-site concentration [Ce], respectively) monitoring on TCI pump device showing sufficient elimination of the drug, the patient still had no signs of recovery of consciousness or even spontaneous breathing. At this point, BIS was checked of 38. Neuromuscular monitoring using train-of-four ratio showed 0.97, which confirmed adequate reversal of muscular blockade. Considering excretion, although there was a history of right renal infarction, the patient's preoperative CT revealed restored perfusion and he showed adequate urine output during operation. And as we used an effect-site pump device, we assumed that a proper amount of drug was administered and that there was no overdose. Therefore, we started narrowing other possible causes of delayed emergence related with nonpharmacological factors. However, ABGA result was also unremarkable in general (Table 1). Hyponatremia was checked; however, ionized sodium concentration of 124 mM was not significantly different from its initial value.

**Table 1:**
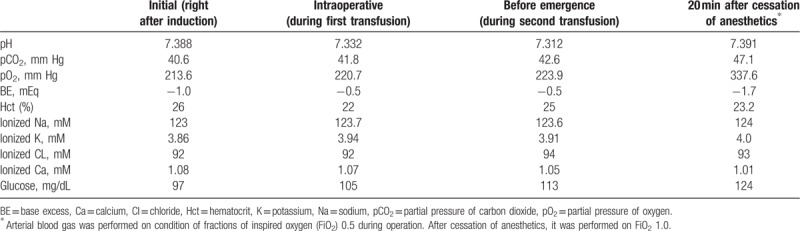
Arterial blood gas analysis of the patient.

**Figure 2:**
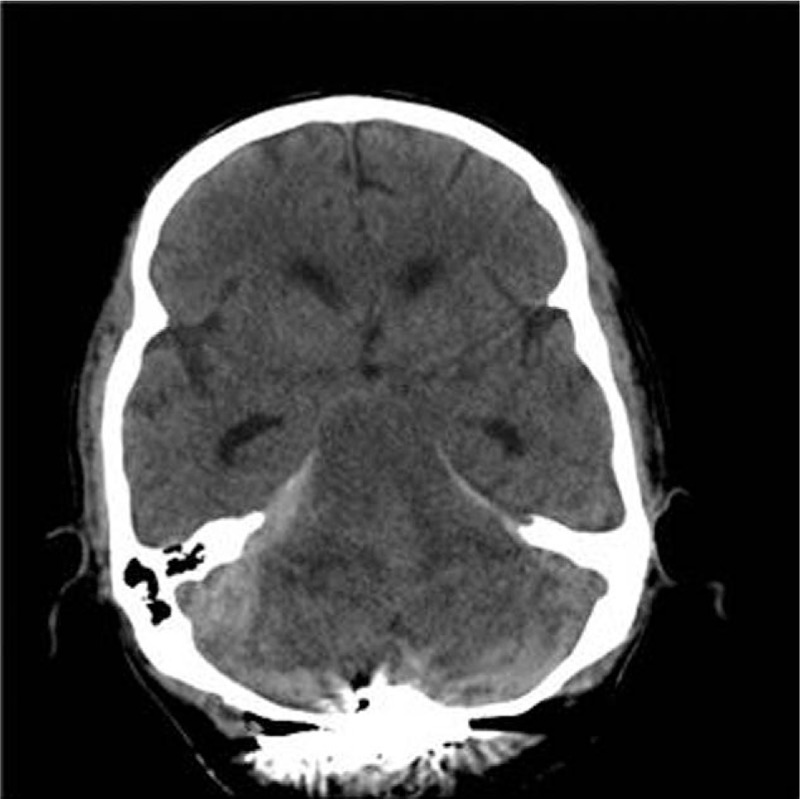
Postoperative brain computed tomography image of the patient showing infratentorial hemorrhage along tentorium cerebelli.

Nevertheless, 20 minutes later, the patient's BP suddenly began to decrease to 86/45 mm Hg, with an HR of 85 bpm. CVP also decreased from 8 to 5 mm Hg. CI, SVV, and ScvO_2_ were 2.5 L/min/m^2^, 18%, and 79%, respectively. Volume resuscitation with a vasopressor was required to restore his vital signs eventually. After the patient received an additional 250 mL of PRBC with support of continuous infusion of levarterenol bitartrate at a rate of 0.03 mcg/kg/min, the patient's BP was restored to 96/54 mm Hg. However, there were still no signs of recovery of BIS or spontaneous breathing. Patient's pupils exhibited dilation, with no response to light. As other factors that may have affected emergence were excluded already, and based on the circumstances, we could expect there could be some bleeding spot which is leaking continuously and responsible for this prolonged emergence; accordingly, the surgeons agreed to examine CT for further checkup. The patient was moved immediately to undergo CT. Infratentorial hemorrhage developing along tentorium cerebelli was revealed, which compressed the 4th ventricle, vermis, and the brainstem (Fig. 2). For exact diagnosis of the reason of this hemorrhage, further image workup of sagittal view using magnetic resonance imaging was required, but we could not get additional information as family of the patient refused more management as they considered his limited original life expectancy. Thus, they also refused the decompression operation to remove the hemorrhage. And the patient expired 5 days later.

## 3 Discussion

Emergence is an exit process from an anesthetized state and its neurobiology is not fully understood. However, there has been increasing interest in comprehending this process over the past decade.^[1–3]^ And we could aware that it is rather a distinctive and active process, not just a passive elimination period of anesthetic agents. Diverse critical complications could occur during emergence. Thus, it is important for anesthesiologists to recognize various conditions of patients which can affect the emergence process, and respond appropriately.

The time to recover complete consciousness is affected by many factors^[4–6]^: anesthetic drug factors, patient factors, surgical factors, and painful stimulation. Emergence from anesthesia can be delayed occasionally, and in most cases, it is caused by residual anesthetic drug-related with overdose.^[4,5]^ Typically, a reduction to 30% of minimum alveolar concentration or decrease of 80% in the effect-site concentration is required for emergence from inhalation or intravenous anesthetic agents, respectively. Time of administration, total dosage, absorption, distribution, excretion, and interactions with co-administered drugs are important for this process. Pharmacological factors do not necessarily mean overdose only, and could be influenced by other patient and surgical factors.

Nonpharmacological causes may result in serious sequelae. Therefore, recognizing the organic conditions of the patient is essential for anesthesiologists. First, for patient factors, we should fully aware preoperatively of the information about patient's age, genetic variations, and preexisting disease, especially which could affect emergence process, such as renal, hepatic, thyroid, or cognitive dysfunction. Other metabolic conditions of the patient that could delay emergence include severe hypo- or hyperglycemia, acidosis, and electrolyte imbalance. Furthermore, we should also consider surgical factors. Unexpected hypotension, cerebral hypoxia, hemorrhage, and embolism could occur during operation and it can end up with prolonged or even failure of emergence. Compared with delayed emergence, failure to awaken is a rare complication. Because of its uncommon incidence and confusion with simple residual drug effect, it would be difficult to recognize a failure of emergence immediately. However, if it occurs, the consequence could be devastating like our case. Therefore, it is important for anesthesiologists to be well-acquainted with those possible causes to differentiate complex conditions from simple delayed emergence with residual drug effect.

To prevent patient's distress when the emergence is prolonged, taking systematic steps to make a differential diagnosis of possible causes seems crucial. First, assessment of vital sign monitoring, anesthetic chart, and surgical field should be preceded before starting emergence process. There we can confirm proper ventilation and circulation of the patient as well as hemodynamic changes, amount and timing of anesthetic drug administered. As Frost reported,^[6]^ patients are expected to be responsive to stimulation within 15 to 90 minutes after the last administration of anesthetic agents. This illustrates how variable the time required for the recovery is, and it remains a difficult challenge to quantify. However, with the help of monitoring Cp or Ce on the effect-site TCI pump device and end-tidal inhalation agent concentration on ventilator, we could estimate the approximate wash out period of anesthetic agents. In case of neuromuscular blocking agent, we can usually expect the timing of recovery by knowing its dosage, last time of administration, and duration. And if recovery is still uncertain, neuromuscular monitoring can be helpful. If an overdose is suspected, we can try antidotes for each anesthetic agent. When sufficient time for wash out of these agents has passed and there is no suspicion of overdose, anesthesiologists should start searching actively for other nonpharmacological factors that may alter mechanisms in the emergence period.

By monitoring vital signs continuously, we can check circulatory or pulmonary events and also measure temperature of the patient. For further investigations of organic conditions of the patient, ABGA can be a very useful tool at this step. We can confirm whether there are metabolic points to correct such as hypoxia, hypercapnia, electrolyte imbalance, acidosis, or even inappropriate blood glucose level. If the undesirable values are checked, they should be treated immediately. Naseem et al reported extremely prolonged unexpected coma which last 2 months after general anesthesia.^[7]^ The only abnormal finding of that case was hypoglycemia. Though it was not a typical case of hypoglycemic coma, the importance and a great risk of nonpharmacologic factors during emergence can be emphasized by this. In most cases, after stabilizing metabolic values in ABGA, no further step is required for awakening patients from general anesthesia. However, if the patient is still not waking up even with unremarkable status through the inspection steps above, like our case, the anesthesiologist should consider further diagnostic approach. Chest x-ray, CT, further blood tests, or neurological examination can be considered depending on circumstances. Uncommon causes such as central anticholinergic syndrome, and even psychogenic coma can be also suspected at this point.^[5,8]^ In our case, the patient showed no significant point to correct even in ABGA test, before his BP suddenly began to decrease. However, because we were on the way of following those steps to rule out other possible causes as emergence being prolonged, we could suspect the possibility of cerebrovascular event.

The etiology of perioperative intracranial hemorrhage is diverse including coagulopathy, high arterial BP, which may damage the vascular wall, and vascular abnormalities. And rare, but even loss of cerebrospinal fluid (CSF) may cause this damage.^[9]^ In our case, the patient already discontinued anticoagulation therapy 10 days before the operation, and laboratory results were unremarkable. We could also exclude sudden changes in BP that may be related to vascular rupture, because we continuously monitored and managed BP through the arterial line.

Remote cerebellar hemorrhage (RCH) most commonly develops after supratentorial procedures.^[10]^ However, RCH after spinal surgery is an unpredictable and a very rare complication.^[11–13]^ Even when it occurs, the onset of neurological deficit is usually delayed for several hours, days, and even months postoperatively, and patients appear neurologically intact while awakening. Therefore, recognition and management, which often require conservative therapy, are usually performed by the neurosurgeon after the operation and it is not well known in the anesthetic field. Despite its extremely rare occurrence, there have been a few literature reports about describing delayed emergence caused by RCH.^[9,13]^ Nakazawa et al reported a patient who could only nod her head in response to verbal commands while exhibiting right-sided hemiplegia. Huang et al. also reported their experience of generalized seizure attack with delayed emergence. Both these cases were caused by RCH after spinal surgery. Reduction in intracranial CSF pressure with dural opening during spinal surgery has been suggested as the most important factor for this complication.^[9–13]^ A profound CSF loss may cause displacement of cerebellum, otherwise known as the cerebellar sag effect. The stretching and occlusion of draining veins may occur, which could result in hemorrhage as a consequence.

In our case, the hemorrhage was developed in infratentorial region rather than cerebellar parenchyma. Still, change of CSF pressure could have affected the damage in a way. However, since the hemorrhage of the patient did not show typical appearance of RCH exactly, we could also consider iatrogenic damage in surgical field of occipitocervical area, related to vascular abnormalities. Small venous injuries could result in slow and gradual hemorrhage. Therefore, it could be difficult to detect those sometimes, even result in delayed hypovolemic shock.^[14]^ This could explain the reason why we or the surgeons could not have noticed the significant sign of hemorrhagic event in both surgical field and anesthetic hemodynamic monitoring before the conclusion of the operation.

There is little that anesthesiologists can do to predict or prevent both RCH and iatrogenic damage in surgical fields. However, it is important to differentiate those conditions from simple residual drug effect for prompt management. Not to overlook these cases, it is essential for anesthesiologists to remain vigilant of following stepwise approach checking every possible factor that may alter the emergence period. It is not wise to take residual drug effect for granted as the routine cause of delayed emergence, neglecting other possibilities. Regardless of the rare incidence, the result of failure to awaken could be devastating when it occurs. There is no universal protocol for emergence period. However, by reporting this case, we suggest that it would be helpful to reconsider how to approach diagnostic steps when the emergence is delayed unexpectedly, especially when the clearance of the anesthetic agent is almost over. And based on that preparation, we could prevent unnecessary complications and achieve better clinical outcomes.

## Author contributions

**Conceptualization:** Ji Hyun Kim.

**Data curation:** Yehun Jin.

**Investigation:** Ji Hyun Kim, Yehun Jin, Seong Wook Hong.

**Writing – original draft:** Ji Hyun Kim.

**Writing – review and editing:** Ji Hyun Kim.
